# Visual Browse and Exploration in Motion Capture Data with Phylogenetic Tree of Context-Aware Poses

**DOI:** 10.3390/s20185224

**Published:** 2020-09-13

**Authors:** Songle Chen, Xuejian Zhao, Bingqing Luo, Zhixin Sun

**Affiliations:** 1 Key Lab of Broadband Wireless Communication and Sensor Network Technology of Ministry of Education, Nanjing University of Posts and Telecommunications, Nanjing 210003, China; chensongle@njupt.edu.cn (S.C.); zhaoxj@njupt.edu.cn (X.Z.); 2 Jiangsu Key Laboratory of Big Data Security & Intelligent Processing, Nanjing University of Posts and Telecommunications, Nanjing 210003, China; luobq@njupt.edu.cn

**Keywords:** motion capture data, pose browse, motion exploration, auto-encoder

## Abstract

Visual browse and exploration in motion capture data take resource acquisition as a human–computer interaction problem, and it is an essential approach for target motion search. This paper presents a progressive schema which starts from pose browse, then locates the interesting region and then switches to online relevant motion exploration. It mainly addresses three core issues. First, to alleviate the contradiction between the limited visual space and ever-increasing size of real-world database, it applies affinity propagation to numerical similarity measure of pose to perform data abstraction and obtains representative poses of clusters. Second, to construct a meaningful neighborhood for user browsing, it further merges logical similarity measures of pose with the weight quartets and casts the isolated representative poses into a structure of phylogenetic tree. Third, to support online motion exploration including motion ranking and clustering, a biLSTM-based auto-encoder is proposed to encode the high-dimensional pose context into compact latent space. Experimental results on CMU’s motion capture data verify the effectiveness of the proposed method.

## 1. Introduction

With the appearance of various motion sensors such as Kinect and motion capture devices, more and more motion capture data are collected and form large 3D human motion databases [1,2]. As a result, how to acquire target motions from repositories has become one of the fundamental problems in the field of computer graphics and computer vision, and involves various applications from traditional computer animation, interactive games to cutting-edge virtual, augmented and mixed reality. The challenges of this task lay in the different magnitude of human skeletons, high dimension of 3D poses and spatio-temporal deformation of homogeneous motions, etc. Over the time, lots of approaches have been suggested to tackle these problems [3,4,5,6,7,8], the majority of which fall into the paradigm of query by example (QBE) of content-based motion retrieval (CBMR).

In QBE, the primary role of the user is that of formulating a query, while the system is given the task of finding relevant matches. The concrete forms of the query example of QBE systems include a single pose with 3D coordinates of joints [3], a motion clip of motion capture data [4], a hand-drawn stroke motion [5], hand-drawn stroke poses [9], etc. They are supposed to explicitly express users’ search intentions. Obviously, it is very hard for users to obtain such query examples. Moreover, retrieving motions based on an explicit query requires users to have a clear perception of what they need in the first place. This may often not be the case, and instead the information need may initially be very vague.

On the other hand, visual browse and exploration in motion capture data take resource acquisition as a human–computer interaction problem [10]. The system applies graphical techniques to visually represent the collection of motions corresponding to the user’s interactive actions, and then the user attains new insights in support of decision making and perception revising after learning the change of visual context. This sequence is repeated until the relevant motions in the visited path can meet the goal. Compared with QBE, it does not require users to provide the query example. It also helps people who are unsure about their goals or unfamiliar with the domain. As a result, visual browse and exploration not only provide a novel paradigm for target search, but also furnish an important interface for users to recognize the overview of the dataset [11].

Besides providing support to online interactive operations such as zoom, pan and selection, there are two necessary components for visual browse and exploration, namely data abstraction and neighborhood construction. In most cases, the segments of motion clips are the ultimate goal for search, but the segment granularity varies with applications from instance to instance. Therefore, individual poses are promoted as an alternative for data organization as they are the smallest unit [10]. Movements consist of continuous poses, and there are a great deal of almost same poses scattering in different movements. The task of data abstraction is to identify such almost same poses and choose their representatives, so the system can fully use the limited visual space. The task of neighborhood construction is to arrange the representative poses in order in the contiguous observation space, so the user can locate the search target quickly through analysis and comparison [12].

Visual browse and exploration have been widely used in content-based multimedia retrieval, including images [13], videos [14] and 3D shapes [15]. At present, the research of organizing motion capture data for browse and exploration is still at the outset stage. The purpose of motionExplorer [10] is the most consistent with us. For pose browse, it iteratively applies KMeans to 3D coordinates of joints to create hierarchical aggregations. The performance of data abstraction and neighborhood construction are entirely dependent on KMeans which is susceptible to the initialization and noise. For motion exploration, it need users to specify both the start and end pose cluster, which is inconvenient and the more desired way is users can promptly perform motion exploration in any pose region they are interested in. Moreover, after data abstraction, the neighborhood poses belong to various motions with different kinds and styles, and it is necessary to provide effective tools including motion ranking, clustering for online exploration when user locating the interesting region of poses. Unfortunately, this is completely overlooked by previous works and obviously hinders the efficiency of target motion search. Nevertheless, classical algorithms such as dynamic time warping [16] for motion distance measure have high computational complexity and cannot meet the requirement of online motion exploration.

**Figure 1:**
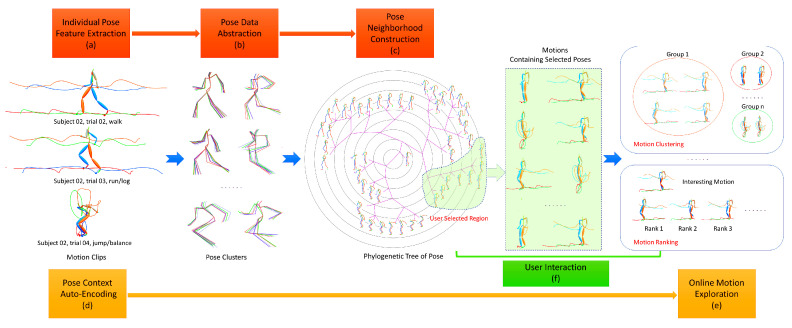
Illustration of the proposed approach for visual motion search. (**a**) With the input 3 motion clips, the individual pose features are extracted for each pose. (**b**) Data abstraction identifies large amount of almost same data and obtains 49 pose clusters. (**c**) Neighborhood construction casts the isolated representative 49 poses of clusters into a structure of phylogenetic tree for pose browse. (**d**) Each pose context is encoded into the compact latent space. (**e**) It is used to support online exploration including motion ranking and clustering after user locating the interesting region. (**f**) Various types of interactive operations such as zoom, pan and selection are available for pose browse and motion exploration.

In this paper, we present a novel approach to organize motion capture data for pose browse and motion exploration. As shown in Figure 1, with the input motion clips, it first extracts individual pose features for each pose (a). Then, it performs data abstraction and obtains representative poses of clusters (b). Next, to construct a meaningful neighborhood for user browsing, it casts the isolated representative poses into a structure of phylogenetic tree (c). Finally, each pose context is encoded into the compact latent space (d) for fast motion exploration (e) after user locating the interesting region when browsing poses. It also supports various types of interactive operations such as zoom, pan and selection for pose browse and motion exploration (f). The main contributions of this paper include:

(1) We present a progressive schema for visual motion search which starts from pose browse, then locates the interesting region and then switches to relevant motion exploration including online motion ranking, clustering.
(2) For data abstraction, it applies affinity propagation to the numerical similarity measure of pose to generate data clusters, the abstracted level of which can easily be consist with human perception by controlling the unique parameter of preference.
(3) For neighborhood construction, it further merges logical similarity measures with weight quartets to represent topological constrains of poses and their reliability, which can produce more reliable neighbors for each pose with global analysis.
(4) For online motion exploration, the high-dimensional pose context is encoded into the compact latent space based on biLSTM, the performance of which matches the classical distance algorithms for time series but with high computation efficiency.

## 2. Related Work

***Motion retrieval.*** The paradigm of query by example (QBE) of content-based motion retrieval (CBMR) draws the most attention of academia in the field of relevant motion search [3,4,5,6,7,8]. Just as content-based image retrieval (CBIR) [17], feature extraction and similarity measure are two most important parts in CBMR. Although the query example of CBIR is commonly accepted as a single image, query example in the QBE systems of CBMR can be found in various forms, such as a single 3D pose [3], strictly aligned motion clip [6,7], slightly misaligned motion clip [18], depending on their different assumptions on the boundaries of motion clip. It can be seen that most of the current CBMR methods impose additional constraints on query examples, regardless the fact that obtaining 3D query examples of motion is hard for users. Our method provides an alternative for CBMR and supports visual motion search with browse and exploration, which does not require users to provide query examples.

***Motion organization.*** The rapid growth of motion capture data requires effective ways and means to organize large collections to meet the needs of various applications. For example, in order to compress the original data and eliminate the spatio-temporal redundancy, Chattopadhyay et al. [19] built the BAP index with intelligent exploitation of the hierarchical structure of human skeleton, and Liu et al. [20] established the index based on piecewise-linear components. To quickly locate the target for motion retrieval, Keogh et al. [21] indexed the motion capture date with R-Tree while Pradhan et al. [22] established the index by using singular value decomposition. For data-driver motion synthesis, Kovar [23] and Min [24] constructed motion maps, in which motion blocks are as nodes and the probability of motion transition are as edges. In this article, we concentrate on organizing the collection of motion capture data in 3D observation space for visual motion search.

***Motion visualization.*** Human motion covers different kinds and styles and involves various visual-interactive applications, where motions together with their parameters need to be visualized for observation, inspection and analysis. Bernard et al. [25] proposed a semi-supervised approach for labelling motion capture data. Although the selected key pose is displayed, but the center is the visual comparison of user-defined labels. On the contrary, the selected pose and its neighbors in our work are situated in the center for the comparison with the intention target. Wagner et al. [26] suggested a knowledge-assisted visual analytics solution for clinical gait analysis. The input data and main part to display are two vertical components of the ground reaction force of feet. Instead, our work needs to show the adjacent 3D poses of motion capture data. Jang et al. [27] suggested MotionFlow, a visual analytics system showing the common pathways of sequences of various motion patterns. The pose clusters in MotionFlow is connected along the time axis, but in our work, the pose clusters are placed only according to their similarity.

***Motion exploration.*** Several visual browse methods have been proposed for motion capture data. Schroeder et al. [28] suggested a trend-centric approach for visual analysis of motion collections. Such approaches are appropriate for the collection whose element movements are homogeneous, while the motions in our method span over multiple categories. Jang et al. [29] adopted joint relative distance to perform interactive hierarchical clustering which enables rapid categorization of similar gestures, and visual investigation of various geometric and kinematic properties. Bernard et al. [10] iteratively applied KMeans to 3D coordinates of joints to create hierarchical aggregations. These two methods only considered single similarity measure and the processes of neighborhood construction were trapped in local optimum. Our preliminary conference work [30] combined several similarity measures for neighborhood construction, but simply treated all the generated quartets as equal importance. In this work, we quantify the reliability of quartets to construct the hierarchy tree with global analysis. Moreover, due to lack of efficient similarity measure for time series, all these methods [10,28,29,30] cannot support online motion exploration for fast visual motion search. In this work, a biLSTM-based auto-encoder is proposed to encode the high-dimensional pose context into compact latent space for online motion exploration.

***Motion encoding.*** When finding the interesting region of poses, users usually need to perform fast online motion exploration such as motion ranking, clustering. However, the computational complexity of classical algorithms for multivariate time series such as dynamic time warping (DTW) [16] is too high to support online exploration. Recently, several motion descriptors based on deep learning technology are proposed. Holden et al. [31] extracted the relative 3D coordinates of joints to form a 2D image and then trained the network using denoising auto-encoder to get the hidden representation. Wang et al. [32] split human body into five channels and used RBM auto-encoder to map motion segments into deep signatures. Sedmidubsky et al. [33] transformed the joint trajectories to 2D image, and then input it into a fine-tuned deep convolutional neural network to extract 4096 feature vector from the last hidden layer. The inputs of these methods are preprocessed motion segments, but in this work, we need to encode the motion context for each pose and we propose a bidirectional Long-Short-Term Memory (biLSTM) [34]-based auto-encoder to fulfill this task.

## 3. Our Method

The pipeline of the proposed approach which supporting 3D pose browse and motion exploration for progressive motion search is shown in Figure 1. The main components include data abstraction with unsupervised affinity propagation to obtain representative poses of clusters, neighborhood construction with phylogenetic tree to organize the similar isolated representative poses to be adjacent with each other, and biLSTM-based auto-encoder to encode the pose context into the compact latent space to support online motion exploration. In this section, each component will be presented in detail.

### 3.1. Data Abstraction

Motion clips usually contain several motions, but the segment granularity varies with applications from instance to instance. Therefore, it is inappropriate to directly organize the segmented motions for browse. The alternative is to organize poses which are the smallest unit of human movements. However, there are large amount of almost same poses in the collection and it is useless to crowdedly display them in the limited visual space. In this section, data abstraction with unsupervised affinity propagation [35] is presented, the purpose of which is to produce a set of representative poses to represent the whole collection.

**Figure 2:**
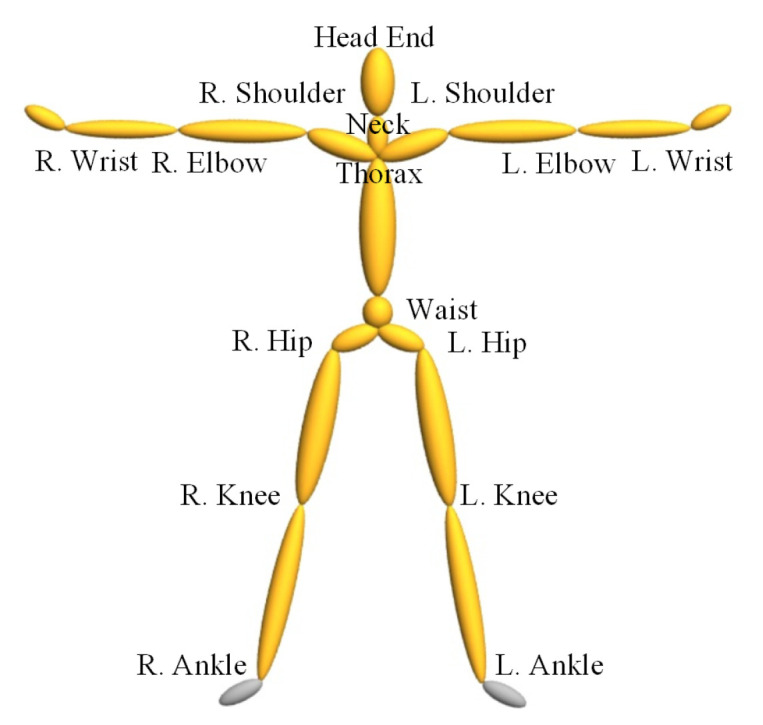
Skeleton model of human pose.

The distance of 3D coordinates of joints is numerical similarity measure and generally used to measure if two poses are very similar to each other. The skeleton models of different motion capture data may be different with each other. In our implementation, we convert these models to a unified one as shown in Figure 2. It contains 16 important joints and is enough to deduce if two poses are very similar. The limb length of different actors may vary greatly, so we need to choose a standard skeleton to normalize different actors’ motions. This is done by replacing each joint offset with the standard one and using the joint rotation to recalculate the 3D coordinates. In the direction normalization step, to make pose always be viewed as head-on, the waist of each pose is shifted to the origin of the coordinate system, and two hips are rotated around the y-axis to make it parallel with x-axis. After normalization, the 3D coordinates of correspond joints of different poses are comparable with each other, and they are extracted and input to the next step, i.e., unsupervised clustering.

Cluster analysis is to group a set of objects in such a way that objects in the same group are more similar to each other than to those in other groups. It is a natural choice for data abstraction and has been applied to many visualization systems. KMeans [36] has been used to perform this task for motion capture data [10]. However, KMeans is heuristic and needs to first specify the group numbers which indeed is difficult to anticipate in advance. In this work, we adopt Affinity Propagation (AP) [35] for data abstraction, which performs clustering by passing messages between data points and has received widespread attention in domains of computer and biological science.

Let $X=\{x_{1},x_{2},\ldots \ldots ,x_{n}\}$ be the set of all poses of the collection of motion clips, $x_{i}\in R^{48}$ is the 3D coordinates of 16 joints. In AP, the responsibility r(i,k) sends from pose *i* to pose *k* and indicates how strongly it favors *k* over other candidate exemplars. The availability a(i,k) sends from pose *k* to pose *i* and indicates to what degree the candidate exemplar *k* is available as a cluster center for pose *i*. The procedure of data abstraction with AP can be summarized as follows:

(1) Calculate the matrix *S* of pose similarity, $s_{ij}=-\max\left(|x_{iv}-x_{jv}\right)\left|\right)$, where v=1,…,16 is joint index.
(2) Set the preference *p* for exemplars, p=median(S)∗δ, δ is the regulatory factor. Run following iterations.
(3) Calculate the responsibility between pose *i* and pose *k*, $r_{t+1}\left(i,k\right)=s\left(i,k\right)-\max_{j\neq k}\left(s\left(i,j\right)+a_{t}\left(i,j\right)\right)$.
(4) Calculate the availability between pose *i* and pose *k*, $a_{t+1}\left(i,k\right)=\min\left(0,r_{t}\left(k,k\right)+\sum_{j\neq i,k}\max\left(0,r_{t}\left(j,k\right)\right)\right)$ and $a_{t+1}\left(k,k\right)=\sum_{j\neq k}\max\left(0,r_{t}\left(j,k\right)\right)$.
(5) Update the responsibility, $r_{t+1}\left(i,k\right)=\left(1-\lambda \right)r_{t+1}\left(i,k\right)+\lambda r_{t}\left(i,k\right)$, where λ is the damping factor.
(6) Update the availability, $a_{t+1}\left(i,k\right)=\left(1-\lambda \right)a_{t+1}\left(i,k\right)+\lambda a_{t}\left(i,k\right)$.
(7) Terminate the loop if the exemplar poses are unchanged for several loops. Otherwise, go to step (3).
(8) Output the exemplar *k* for pose *i* as max(a(i,k)+r(i,k)).

AP is a deterministic algorithm and the granularity of data abstraction can be easily controlled by adjusting the threshold preference *p*. If *p* is too high, dissimilar poses may fall into the same cluster. On the contrary, if *p* is too low, very similar poses may spread over different clusters. Empirically, the same joints in a cluster should not exceed half of the forearm for human beings are very familiar and sensitive to the position of the limbs. In our experiments, δ is used to adjust *p* and it is set to 0.20 for CMU’s motion capture data. As denoted in step 1, we calculate the distance of two poses based on the max instead of average difference of two corresponding joints, which is critical to prevent poses from being drastically different with others of the same cluster in some joints.

**Figure 3:**
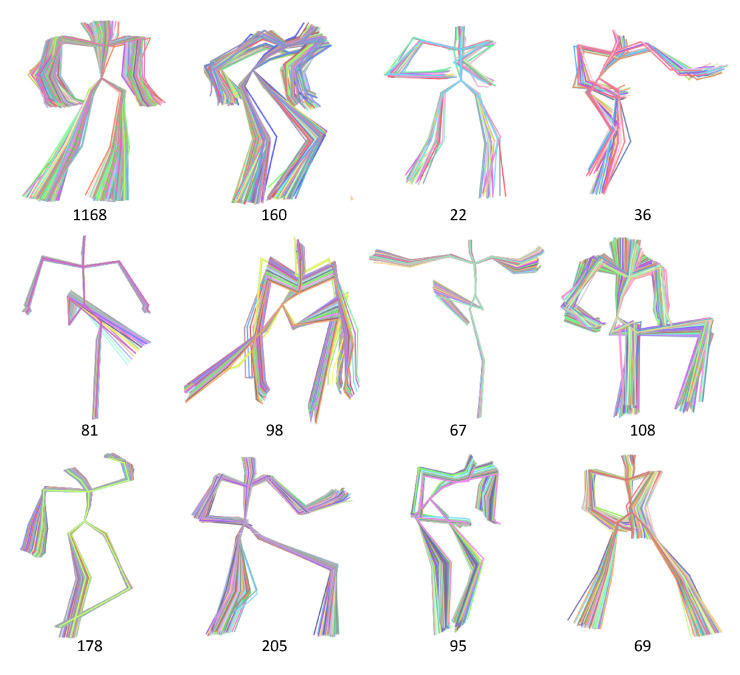
Examples of data abstraction with unsupervised clustering AP.

Some examples of data abstraction are shown in Figure 3, at the bottom is the number of poses in each cluster, ranges from 22 to 1168. The granularity of the results is commonly approved by users, while a fair number of poses need not to be shown by bypassing their direction, skeleton magnitude and motion context. We have implemented the proposed method on the dataset containing about 43 thousands poses. When the scalability of AP needs to be extended for huge data, we refer to the distributed version based on MapReduce [37]. The representative of each cluster is selected from actual poses, they will be input to neighborhood construction described in the next section.

### 3.2. Neighborhood Construction

The result of data abstraction with unsupervised clustering is isolated pose clusters and their representatives. The task of neighborhood construction is to arrange the representative poses in order in the contiguous observation space, so the user can locate the search target quickly through analysis and comparison. Traditional method [10] achieved this goal by iteratively applying KMeans to 3D coordinates of joints to create a hierarchical tree. However, it only considered the numerical similarity measure and the performance is entirely depend on KMeans which is susceptible to the initialization and noise. In this section, we further merge logical similarity measures with weight quartets and cast the collection of isolated representatives into a structure of phylogenetic tree with quartet Max-Cut optimization.

#### 3.2.1. Feature Extraction

The representation of 3D coordinates of joints adopted in data abstraction is a numerical similarity measure and good at measuring if two poses are very similar to each other. Over the past few years, several logical measures have been lodged to perceive the perceptual similarity between poses [3,4,38]. In general, these logical measures take points, lines and planes formed by joints as the geometry elements, and use the set of angle and distance between geometry elements to measure the similarity between poses.

**Figure 4:**
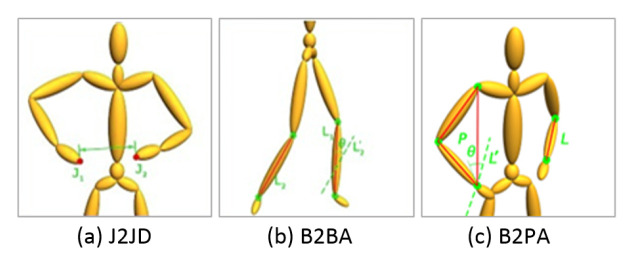
3 types of relative geometric features.

In this work, 3 types of relative geometric features (RGF) are further used as the metric to calculate the perceptual pose similarity, including Euclidean distance of two joints, intersection angle of two bones, and intersection angle of a bone and a plane (call them J2JD, B2BA, B2PA respectively in the following), which involve the measures of distance, angle between geometry elements of point, line and plane. A demo illustration of these 3 types of RGF is shown in Figure 4, sequentially represents Euclidean distance between two hands, angle between two cruses, and angle between left forearm and plane composed by right wrist, right shoulder and right elbow. More types of perceptual similarity metric of poses also can be added, but currently we find these 3 types of RGF with 3D coordinates of joints are enough to get high quality neighborhood through our experiments.

**Figure 5:**
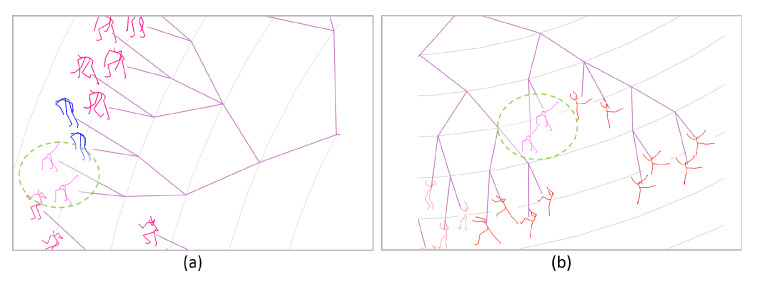
Part of two phylogenetic trees, (**a**) generated with 3D coordinates of joints, (**b**) generated by further merging 3 types of RGF.

The left of Figure 5 illustrates the part of phylogenetic tree generated with only the measure of 3D coordinates of joints, while the right is the result of further merging 3 types of RGF (the whole tree is shown in Figure 17). It can be seen that in the left figure, the two poses enclosed by circles with the dotted green line are improperly placed with its neighbors. As shown in the right of figure, these two poses achieve more reasonable arrangement with the assistance of 3 types of RGF.

#### 3.2.2. Weight Quartet Generation

**Figure 6:**
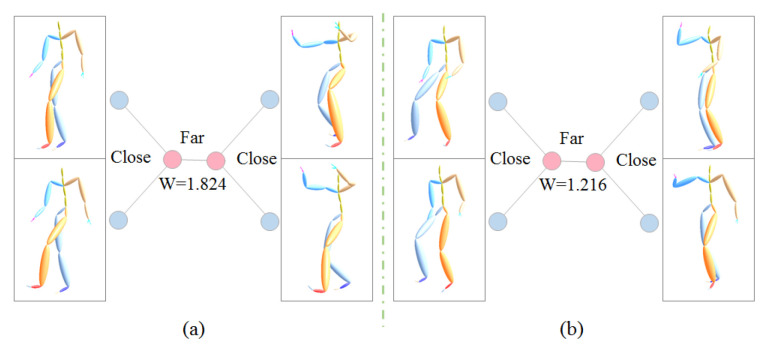
Two quartet samples of poses. (**a**): 1.824, (**b**): 1.216.

One common way to merge these different types of features is by stitching their feature vectors and then uses the traditional bottom-up or top-down algorithms to construct a tree. However, different features are in different metric space and the process is easily trapped in local optimum. Instead, we generate a subset of weight quartets with each type of feature, and merge these subsets to construct a phylogenetic tree by using the Max-Cut optimization. A quartet consists of 4 poses and it is the smallest unit of topological constraint. As shown in Figure 6, poses in the left side or right side are similar to each other. In contrast, poses in different side are dissimilar to each other.

**Figure 7:**
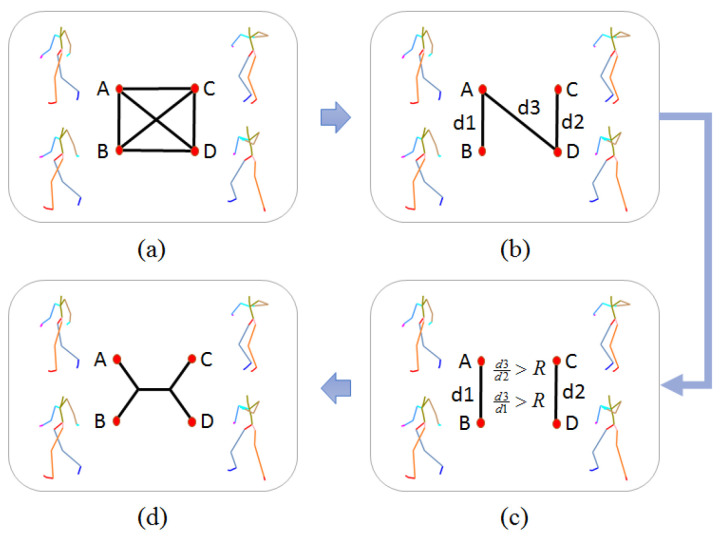
Process of obtaining reliable quartets.

Figure 7 illustrates the steps of generating a quartet with a given set of poses and one type of feature. (a) Select pose A, B, C, D from the set as 4 nodes, and connect these nodes with edges, the weight of each edge is the Euclidean distance of two connected nodes with the given type of feature. (b) Remove the largest 3 edges, then designate the 4th largest edge d3 as the bridge and the smallest two edges as d1 and d2 respectively. (c) With the given threshold *R*, check if d3/d1>R and d3/d2>R. (d) If it fulfills the condition, a reliable quartet with its topological constraint is defined.

Threshold *R* controls the number of quartets and will be discussed in Section 4.2.3 of evaluation. It is obvious that the reliability of quartet is changed with the ratio of bridge to edge (d3/d1 and d3/d2). The larger the ratio is, the more reliability a quartet will be, and it is necessary to quantify the importance of quartets for the Max-Cut optimization of building the phylogenetic tree. For each pose, we sort its *K* neighbors based on the distance of the specific type of feature, *K* is set to 30 which is nearly the number of poses that can be observed in the screen. Suppose $O_{i2j}$ is the rank position of pose *i* in the neighbors of pose *j*. $O_{\hat{AB}}=1/2\left(O_{A2B}+O_{B2A}\right)$ is the average rank position of pose A and B in the neighbors of each other. Similarly, $O_{\hat{CD}}=1/2\left(O_{C2D}+O_{D2C}\right)$ is the average rank position of pose C and D in the neighbors of each other. If d3/d1>R and d3/d2>R, the quartet is reliable and the weight of which is defined as

(1) $$w=\left(1-\frac{d1}{d3}\right)*\lambda^{O_{\hat{AB}}}+\left(1-\frac{d2}{d3}\right)*\lambda^{O_{\hat{CD}}}.$$

Here, λ is the decay factor and the empirical value is 0.95.

**Figure 8:**
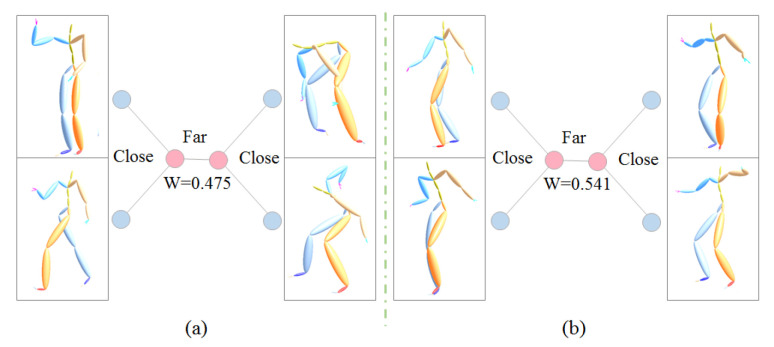
Example quartets with small weight, (**a**): 0.475, (**b**): 0.541.

The weight of a quartet is determined by the ratio of the inner distance to the bridge distance, which is fine-tuned by the average rank position of neighbor order. The smaller ratio of inner distance to the bridge and the higher rank position in the neighbors of each other are, the more reliability the quartet is. By this means, we not only have a limited number of most reliable quartets, but also have enough quartets with reliability at many levels, which will benefit the algorithm of building the phylogenetic tree with subtle hierarchies. In Figure 6, by using B2BA feature representation, the weights of the left and right quartets are 1.824 and 1.216 respectively. Figure 8 shows two example quartets with relatively small weight.

#### 3.2.3. Phylogenetic Tree Construction

For each type of feature, a set of quartets with their weights are obtained. We then combine all these quartets to build a phylogenetic tree and satisfy the maximum number of topologies. This is the maximum quartet consistency problem and can be solved both heuristically and exactly. However, exact methods cause huge computing complexity. For this reason, we adopt a heuristic method, weight Quartet Max-Cut algorithm (WQMC) [39] to build a phylogenetic tree. WQMC first embeds poses into a 3D sphere based on the topological information of the quartets, then recursively partitions the set of poses in a top-down manner.

**Figure 9:**
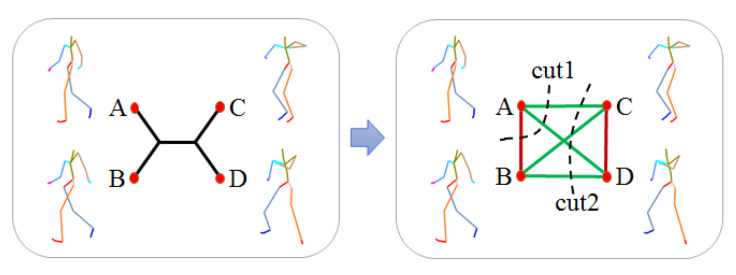
Partition a quartet into groups, the quartet has 6 edges, the red are 2 bad edges and the green are 4 good edges for cutting.

As shown in Figure 9, after partition, pairs A, B and C, D are expected to be in a separate group. To achieve this, WQMC denotes the edges between A, B and between C, D as bad edges, and all other four edges as good edges. A good partition is one that cuts through as many good edges as possible and as few bad edges as possible. As can be seen in Figure 9, cut 2 is better than cut 1 as no bad edges are included in it. Thus, the optimized partition is obtained by maximizing the following expression:

(2) $$\sum_{e(i,j)\in G}d\left(i,j\right)-\alpha \sum_{e(i,j)\in B}d\left(i,j\right).$$

*G* and *B* is the set of good and bad edges from all quartets respectively, d(i,j) is Euclidean distance between the embedded vertex $v_{i}$ and $v_{j}$ representing pose *i* and pose *j*, and α is a scalar weight. Under WQMC, every edge in *G* or *B* is first assigned the weight of the quartet it belongs to, and then the algorithm recursively looks for a cut that maximizes the ratio between the total weight of good edges and bad edges of the resulting subsets. All the found cuts are used to build the final phylogenetic tree, where every cut defines an edge in the construction. For a more comprehensive overview of WQMC, we refer the readers to [39].

The phylogenetic tree is a no root tree, and it can satisfy the maximum number of weight quartets. Figure 1c shows the final phylogenetic tree for pose browse with the input 3 CMU’s motion clips of subject 02, i.e., 02, 03 and 04 trail. It can be seen that similar poses are adjacent with each other, and this similarity decreases with the increasement of the edge count between them. As a result, the user can locate the target pose quickly by tracing the continuous change of poses in the local region.

### 3.3. Pose Context Auto-Encoding

When browsing poses in the phylogenetic tree, the user can quickly locate the interesting region and needs to perform further motion exploration for ultimate motion search. The demo illustration is shown in Figure 1c. The shallow green irregular region in the phylogenetic tree is enclosed by the user when he or she wants to further investigate the relevant motions. According to the trace of data abstraction, the system then provides the segments of motion clip whose poses or their cluster exemplar poses belong to the enclosed region, shown in the right side of the region. For different motions have a certain probability of sharing the similar transitional poses, these segments cover not only different phases of motions, but also different kinds of motions. Obviously, carrying out further comparison with these chaotic motions is a time-consuming and labor-intensive task for the user, and it is necessary to provide effective tools such as motion ranking, clustering for online exploration.

Motion distance measure is the fundamental unit for motion exploration. 3D Human motion is a type of multivariate time series data and there are nonlinear distortions between them. As a result, traditional measures such as dynamic time warping (DTW) [16] calculate the distance between any pair of different poses of two motions and adopt elastic matching to calibrate the frame-level distortions. Consequently, the computational complexity is too higher to perform online motion exploration. For example, the time complexity of DTW is O(m∗n∗p), where *m*, *n* are the length of two motions and *p* is the length of vector representing a pose. Moreover, such measures are focus on motion difference in detail, and hard to capture the overall similarity of different motions. Recently, several motion descriptors based on deep learning technology are proposed [31,32,33], but in this scenario, the input is the motion clip without semantic segmentation and we need to encode the context for each pose.

**Figure 10:**
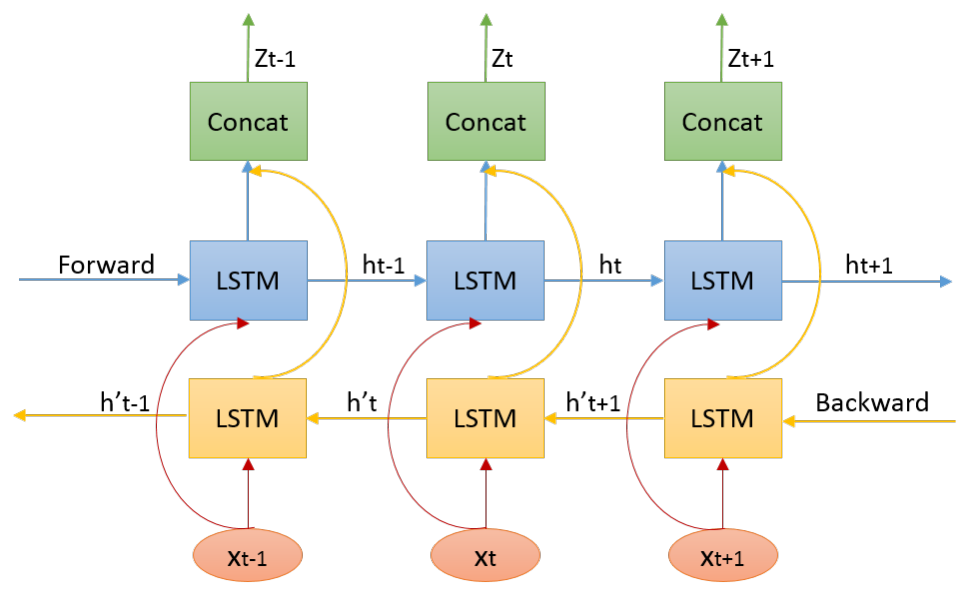
BiLSTM-based encoder for pose context.

In this work, we propose a bidirectional Long-Short-Term Memory (biLSTM) [34]-based auto-encoder to fulfill this task. The encoding network is shown in Figure 10. We adopt LSTM for the non-linearity recurrent to encode the hidden state of the observations for its ability to learn long-range dependencies and stable dynamics. Furthermore, the bidirection enables us to encode pose context based on both previous history and future observations. For an input motion clip, at each time step *t*, pose $x_{t}$ simultaneously inputs to the forward and backward LSTM network and updates their internal states. Then the updated forward and backward hidden states are concatenated with the nonlinear function as the latent representation of pose context. In our implement, we only use one LSTM layer and the number of LSTM unit is set to 512.

**Figure 11:**
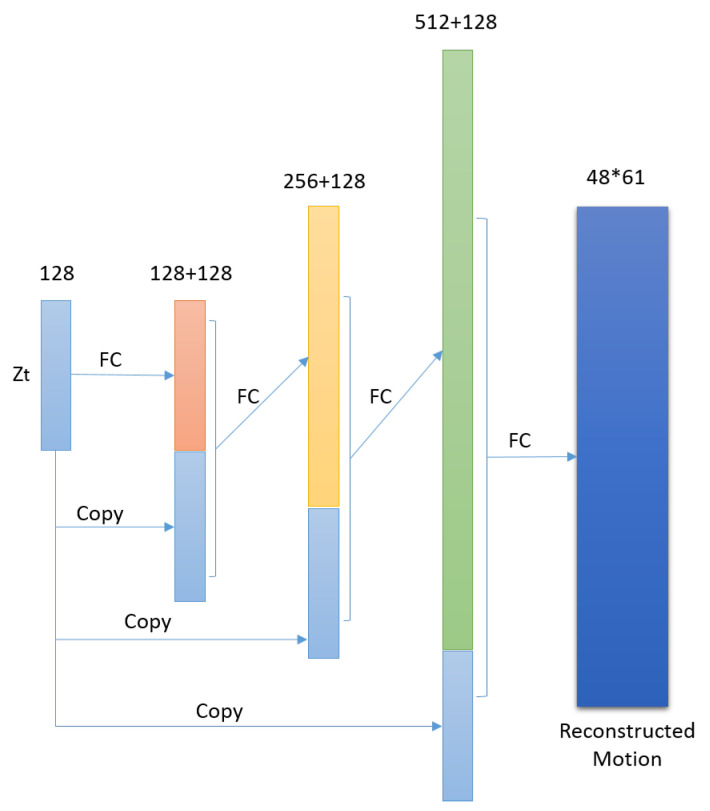
Corresponding decoder for pose context.

For each time step *t*, notice that the decoding target is not $x_{t}$ but the context motion which will be entitled to current pose. Previous 30 poses and future 30 poses are intercepted as the context motion for each pose, which duration is about 2 seconds and covers most actions. The decoding network is not based on LSTM as traditional LSTM auto-encoder. The reason is the latent representation of current $x_{t}$ can recovery the context motion with the aid of its neighbors. The decoding network is shown in Figure 11, the main components of which include copy, concatenation and full connection. The purpose of copy and concatenation is each layer can profit from the latent representation $z_{t}$ directly and make the learning progress faster.

The above auto-encoder only considers the one-to-one pose context reconstruction, which may just learn to perform the identity function without extracting useful representations for similarity measure. Movement of human body is the process of continuously changing poses, so we have reasons to deduce that the encoded variables of two continuous poses of motion in the latent space should be very close to each other. Based on this observation and to preserve the latent geometric information for motion distance calculation, a special similarity constraint is added to loss function.

(3) $$l\left(z_{t-1},z_{t}\right)=\left|\right|z_{t-1}-z_{t}{\left|\right|}^{2}$$

**Figure 12:**
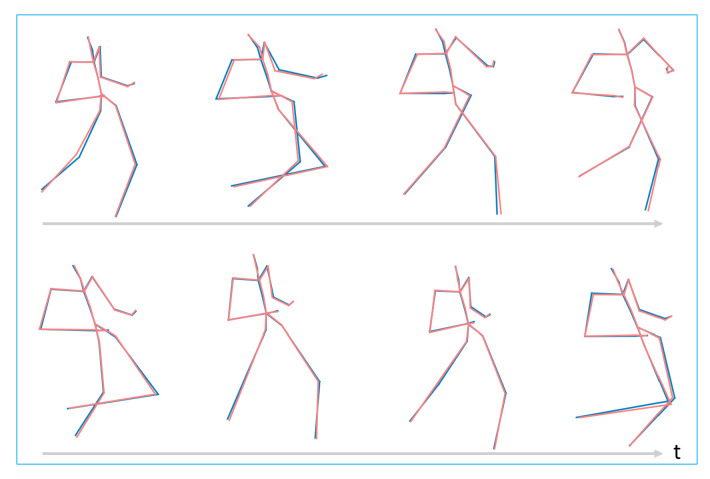
Example reconstructed CMU’s motion of trail 10 of subject 142, frame range from 290 to 509, the walk style is *lavish*. The red line is the original pose, while the blue line is the reconstructed one.

The training is implemented with this joint supervision of the motion reconstruction error and Euclidean distance on latent space of adjacent poses, and the detail of which will be present in Section 4.3. Examples of the original motion and reconstructed motion are shown in Figure 12. The motion is CMU’s motion capture data, trail 10 of subject 142, frame range from 290 to 509, the walk style is *lavish*, and the red line is the original pose in the motion, while the blue line is the reconstructed one. The average deviation is about 0.2 per joint. The offset from left hand to left forearm is 3.6, so there is very little difference between the original context motion and reconstructed motion.

Vector *z* encoded the context motion for each pose. Consistent with the similarity constraint, we use Euclidean distance of *z* as motion distance measure, the time complexity of which is $O(p^{\prime })$, where $p^{\prime }$ is the length of vector *z*, i.e., 128. As mentioned before, the complexity of classical DTW for motion data is O(m∗n∗p), namely O(61∗61∗48) in this case. It is obvious that the proposed Euclidean distance measure on latent space is more efficient and can support the user to perform online further motion exploration such as motion ranking and clustering for ultimate motion search. The proposed motion similarity measure enables us to adopt widely used KNN and AP for this purpose, and the user also can adjust a few parameters of these algorithms according to their online analysis.

## 4. Experiments and Evaluations

### 4.1. Evaluation of Data Abstraction

We carry out data abstraction with AP clustering on 3D coordinates of joints as described in Section 3.1. To evaluate the performance of the proposed method, we compare AP with the representative clustering algorithm KMeans and the method of splitting clusters based on the distance threshold [40] (call it Splitting for short in the following). KMeans has been proposed in motionExplorer [10] to perform data abstraction for motion capture data. The second method also has been broadly used in the key frame extraction and data abstraction. It is chosen to compare with the proposed method for both are deterministic and need only one parameter.

We adopt Davie-Bouldin index (DBI) as a metric for evaluating these three clustering algorithms, which has been widely used and is independent with the data as well as the algorithm. Let *N* be the number of clusters, $A_{i}$ be the centroid of $C_{i}$ and $T_{i}$ be the size of the cluster *i*. $S_{i}$ is a measure of scatter within the cluster and is calculated by

(4) $$S_{i}=(\frac{1}{T_{i}}\sum_{j=1}^{T_{i}}|x_{j}-A_{i}{{|}^{p})}^{1/p}.$$

Then DBI is defined as

(5) $$DBI=\frac{1}{N}\sum_{i=1}^{N}\max_{j\neq i}\frac{S_{i}+S_{j}}{\left|\right|A_{i}-A_{j}{\left|\right|}^{1/p}}.$$

**Figure 13:**
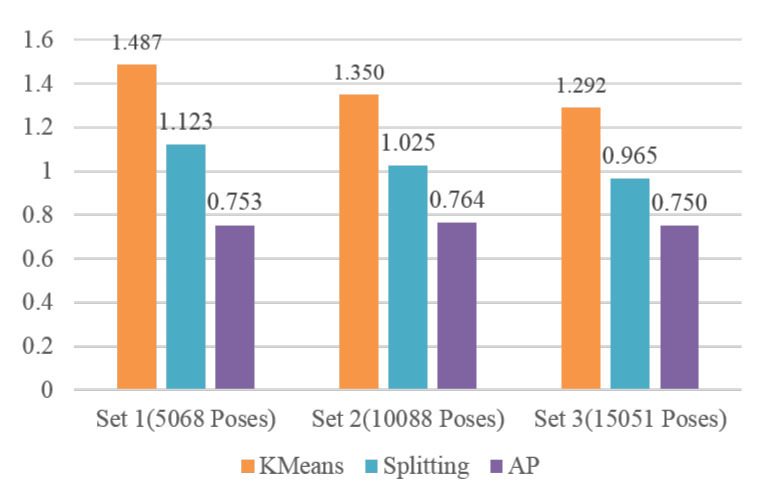
DBIs of KMeans, Splitting and AP on three sets.

We selected three sets of motion clips from CMU’s motion capture database. The first set is composed of 5 clips from 01-01 to 01-05, where the first 01 is the subject ID and second 01 to 05 represent the motion clip index of subject 01, the total number of poses is 5068. The second set is composed of 10 clips from 01-01 to 01-10, the total number of poses is 10,088. The third set is composed of 20 clips from 01-01 to 01-14 and 02-01 to 02-06, the total number of poses is 15,051. For fair comparison, we first applied AP to each set and obtained 970, 2115 and 3515 clusters, respectively. This three numbers were input into KMeans as to specify the cluster number required by the algorithm. We also adjusted the distance threshold for Splitting to generate the same number clusters. The DBIs of three methods on three sets are given in Figure 13.

DBI is the smaller the better. From Figure 13, it can be seen that the DBI of KMeans is the biggest among three methods and its performance is the worst. Splitting method is better than KMeans. AP clearly outperforms KMeans and Splitting method in all three sets. Notice that the DBI of AP is only about half of the KMeans, 0.753 vs. 1.487 in set 1, 0.764 vs. 1.350 in set 2, and 0.750 vs. 1.292 in set 3. The main reason is KMeans is a greedy method and the result of which often falls into the trap of local optimum. Analogously, splitting method only considers the current input data and does not adjust the clusters obtained before. Conversely, with the global optimization and by message passing between all points, AP obtains the better performance for data abstraction.

The time complexities of Splitting, KMeans and AP are O(n), O(n∗T) and O(n∗n∗T) respectively, where *n* indicates the total number of poses need to be clustered and *T* is the number of iterations. While AP has better performance for data abstraction, the complexity of which is quadratic to the number of poses. Obviously, the computation cost is the disadvantage of AP, yet data abstraction is an offline phase. When the scalability of AP needs to be extended for very huge data, we recommend the distributed version of AP based on MapReduce [37]. Another practical alternative is Partition Affinity Propagation [41], which is an expansion of AP and has achieved quite good results on the large-scale datasets, but with much faster speed.

### 4.2. Evaluation of Neighborhood Construction

#### 4.2.1. Criteria

**Figure 14:**
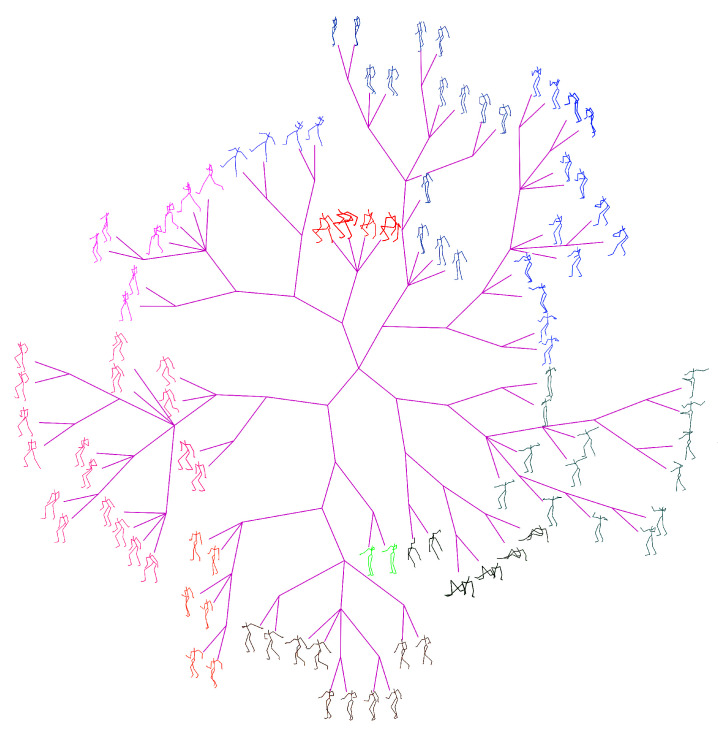
Ground-truth tree of Set #1, manually organized and contains 100 center poses of clusters.

**Figure 15:**
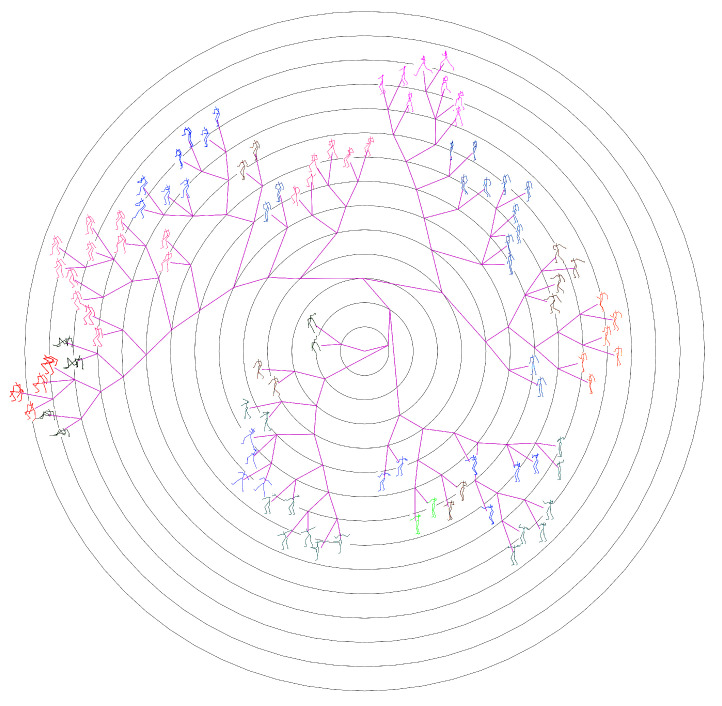
Phylogenetic tree of Set #1 generated by our method.

**Figure 16:**
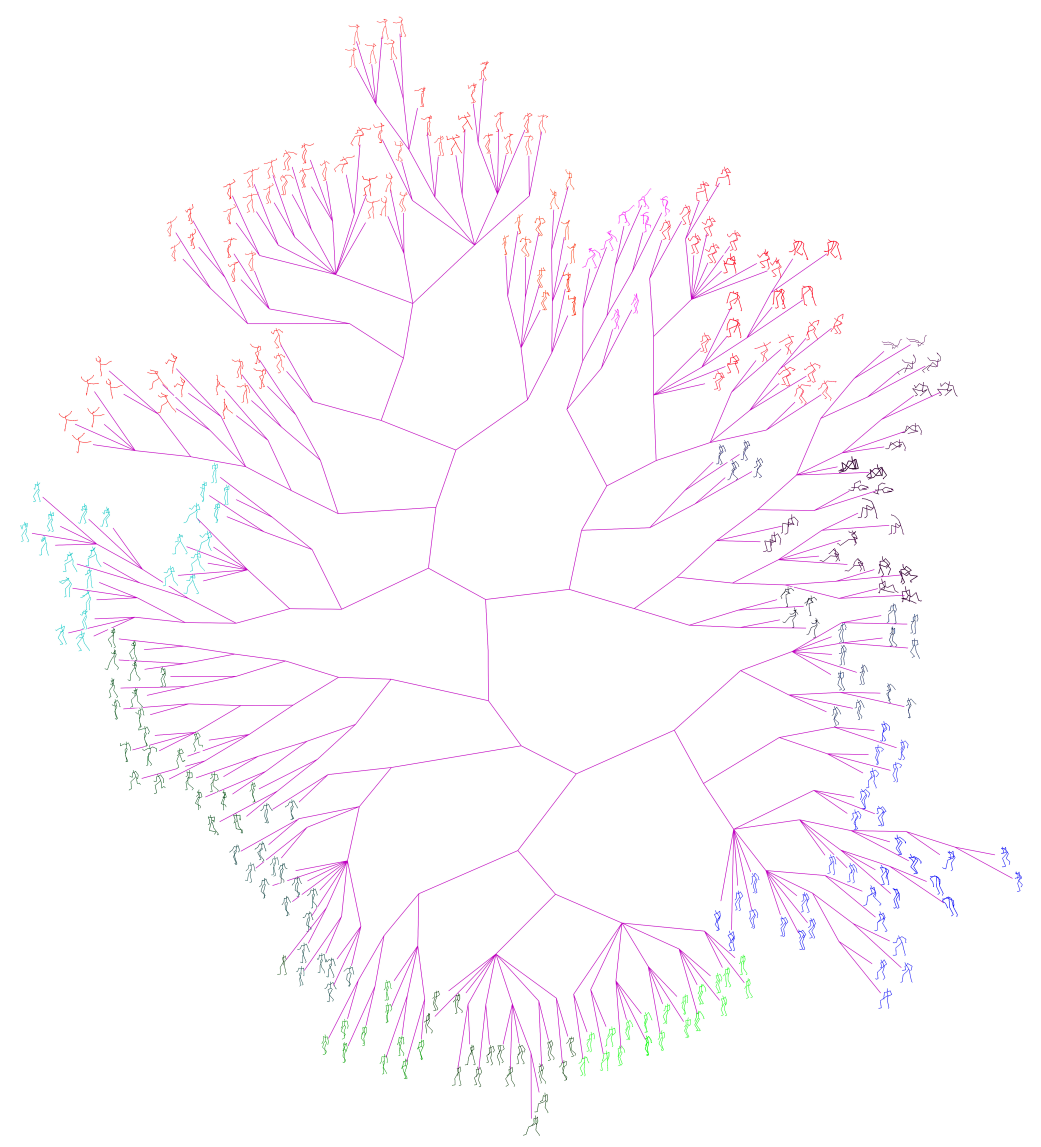
Ground-truth tree of Set #2, manually organized and contains 300 center poses of clusters.

**Figure 17:**
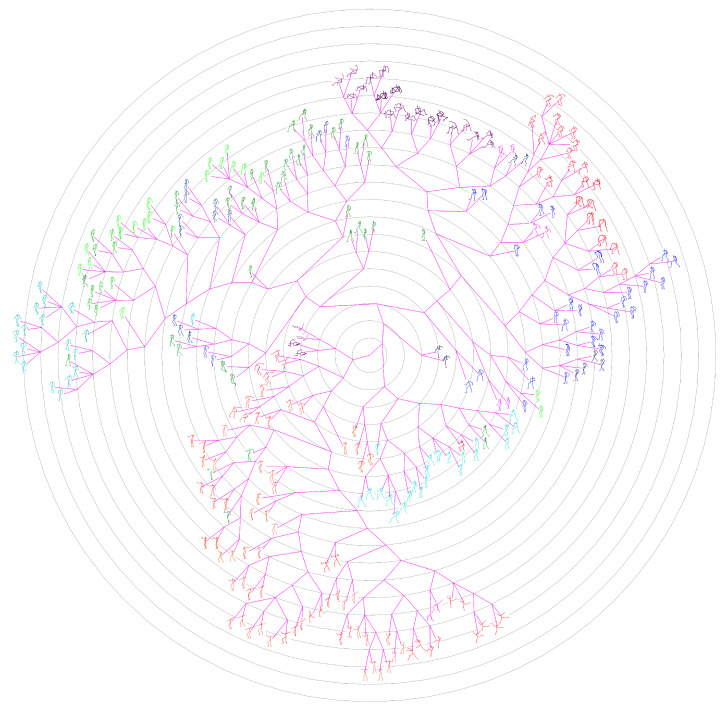
Phylogenetic tree of Set #2 generated by our method.

The ground-truth trees are needed to evaluate the performance of different approaches for neighborhood construction. For this purpose, we randomly selected the representative (center) poses of clusters generated by performing the data abstraction with AP on CMU’s motion capture data, and formed set #1 and set #2 which contains 100 and 300 poses respectively. Then we developed a labelling tool and the ground-truth tree on each set was manually constructed by 10 participants. The poses in the set are allocated to participants averagely. In the first loop, the participants added their allocated poses to the tree one by one, and formed an initial tree. In the next loops, the participants adjusted the location of their allocated poses and structure of the tree one after another. This procedure continued until all participants have consented the labelled results. Figure 14 shows the manually labelled tree of set #1, while Figure 15 presents the tree of set #1 generated by our method. Figure 16 and Figure 17 show the corresponding trees of set #2.

The representations of adjacent neighbors of different approaches may be different, so a compatible evaluation criterion is needed. Given a pair of poses *p* and *q*, the rank distance of *q* to *p* with algorithm *a* is defined as the number of poses in the whole dataset which are nearer to *p* than *q*, i.e.,

(6) $$r_{a}\left(p,q\right)=count\left(v\right),v\;s.t.\;d_{a}\left(p,v\right)<d_{a}\left(p,q\right).$$

Different algorithms for neighborhood construction define different visual spaces. $d_{a}$ is the distance of two poses in the visual observation space with the given algorithm *a*. For a tree, *d* is the edge count between two nodes of poses. For a scatter plot, *d* is the Euclidean distance between two points of poses. When considering *s* nearest neighbors for each pose, the average deviation *D* of *a* from the ground-truth gt for neighborhood construction is

(7) $$\begin{array}{cc} D=\frac{1}{2N} & \sum_{i=1}^{N}(\frac{1}{K}\sum_{k=1}^{K}|r_{gt}\left(p_{i},q_{k}\right)-r_{a}\left(p_{i},q_{k}\right)|+\frac{1}{K^{\prime }}\sum_{k^{\prime }=1}^{K^{\prime }}|r_{a}\left(p_{i},q_{k^{\prime }}\right)-r_{gt}\left(p_{i},q_{k^{\prime }}\right)\left|\right). \end{array}$$

where pose $q_{k}\;s.t.\;r_{gt}\left(p_{i},q_{k}\right)<s$ and $q_{k}^{\prime }\;s.t.\;r_{a}\left(p_{i},q_{k^{\prime }}\right)<s$. *N* is the total number of poses in the data set. The first part of the formula takes the total *K* pose pairs in ground truth which satisfies $r_{gt}\left(p_{i},q_{k}\right)<s$ as the criterion, while the second part of the formula takes total $K^{\prime }$ pose pairs in neighborhood representation *a* which satisfies $r_{a}\left(p_{i},q_{k^{\prime }}\right)<s$ as the criterion. This means we not only expect the neighbors in the ground truth are still the neighbors in approach *a*, but also expect the neighbors in approach *a* are still the neighbors in ground truth. By this means, we can discern the approaches getting high scores by degenerating to place most poses as the nearest poses for each pose. Formula (7) will be used as the criterion in the following for evaluation.

#### 4.2.2. Comparison with the State-Of-Arts

Figure 15 and Figure 17 present the phylogenetic trees corresponding to set #1 and #2 generated by our method WQMC. We compare it with KMeans [36], PCA [42] and neighbor joining (NJ) [43] three traditional approaches for visual browse and exploration. KMeans was adopted by MotionExplorer [10] to iteratively partition the poses to form a hierarchical tree. PCA is a parameterless method for dimension reduction and is often used in the visualization system [44]. NJ is a classical method to create a phylogenetic tree by bottom-up clustering. Moreover, our preliminary conference work [30] adopted QMC for neighborhood construction and is also compared. Just like MotionExplorer [10], we directly input 3D coordinates of joints into KMeans, and the same format is used for PCA. We use 3D coordinates of joints to calculate the distance matrix for NJ. For QMC and WQMC, 3D coordinates of joints and 3 types of RGF are adopted as feature representations. The iteration of KMeans is always set to 10,000 to obtain the hierarchical trees.

**Figure 18:**
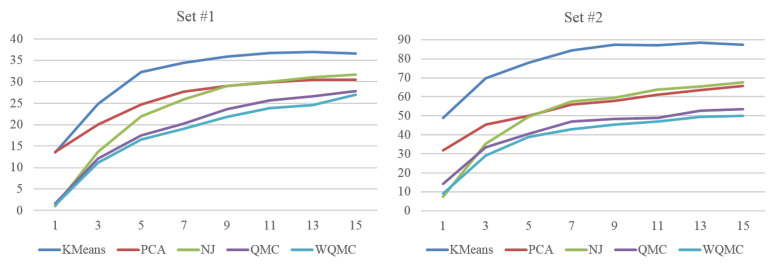
Average distance of *s* nearest neighbors of five approaches on set #1 and set #2.

Figure 18 shows the performance of neighborhood construction of five approaches on set #1 and #2. The *X* axis in Figure 18 is the number of nearest neighbors *s*, and *Y* axis is the average distance *D* as defined in Formula (7). The criterion is strict for if a pose is misplaced, it not only causes the errors of its ground-truth adjacent neighbors, but also adds the errors to the poses mistakenly adjacent to it. So the average distance increases quickly with the sample size and number of nearest neighbors.

Although there is a little difference in the performance among two sets, the tendency is consistent in general. The performance of KMeans is the worst among all. For example, when s=5, the average distance of KMeans is 32.30 on set #1 and obviously larger than other methods. The reason may be due to KMeans is sensitive to the initialization, noise and data balance, and the error is multiplied by each partition. Ultimately, we could speculate that the splitting strategy combining with KMeans is not an effective way for neighborhood construction. PCA projects the data into the main components, but with linear transformation, the two largest components corresponding to *X* and *Y* axis of 2D visual space inevitably loss some important information when dealing with the complicated human motion data. When *s* is very small, NJ even obtains the best performance, but as a local optimization algorithm, it gets worse and worse quickly and even be inferior to PCA. QMC can merge several similarity measures with quartets to represent topological constrains of poses. With the global analysis, the performance of QMC is superior to that of KMeans, PCA and NJ. WQMC inherits the merits of QMC, but further assigns weights to quartets to reflect their different reliability. It achieves the best performance among five approaches.

**Figure 19:**
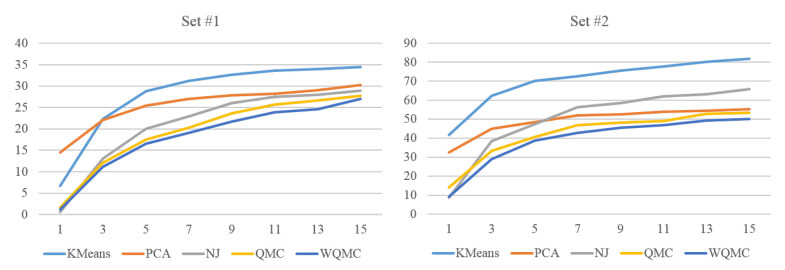
Average distance of *s* nearest neighbors of five approaches on set #1 and set #2, with same feature representations.

In the above experiment, KMeans, PCA and NJ use 3D coordinates of joints as feature representation, while QMC and WQMC additionally adopt 3 type of RGF. One may own the performance difference to different feature representations. For fair comparison, we first normalize each type of feature and scale it to range 0 and 1, then merge these 4 types of features into one vector and input it to KMeans and PCA. For NJ, we use the merged vector to calculate the distance matrix. Figure 19 shows the performance of neighborhood construction of five approaches on set #1 and #2 using the same feature representations. The proposed method still outperforms all other methods on both two sets. Taking set #2 as instance, when s=1, the average distance of WQMC is 9.12 while which of KMeans is 41.77. When s=5, the average distance of WQMC is 38.77 while which of KMeans is 70.22, our method has the clear advantage over MotionExplorer [10].

#### 4.2.3. Self Comparisons

**Figure 20:**
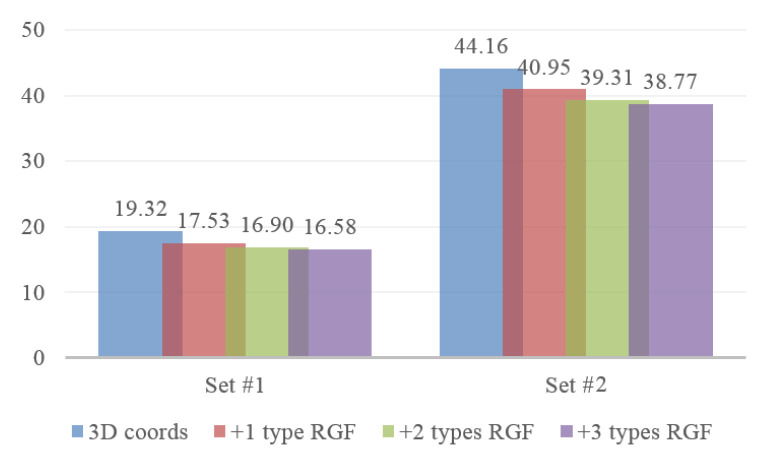
Average distance of combining different number of types of RGF with 3D coordinates of joints on set #1 and set #2, *s* is 5.

We adopt WQMC through quartets to further merge logical similarity measures for neighborhood construction. To verity the strength of the proposed approach, we compare the quality of the resulting phylogenetic trees generated with the subsets of 3 types of RGF and 3D coordinates of joints. The set of weight quartets created further using *j* types of RGF is denoted by $I_{j}$. For example, when *j* is 2, the number of combinations is $c_{3}^{2}$, so there are 3 elements in $I_{2}$. For each element in $I_{j}$ and quartets generated from 3D coordinates of joints, we construct the tree with the Max-Cut optimization and the performance of which is calculated according to Formula (7). Figure 20 presents the results of different combinations on set #1 and #2 when the number of nearest neighbors *s* is 5. It indicates that the performance of our method is enhanced by further combing RGFs with 3D coordinates of joints. However, the growth becomes smaller and smaller with more and more types of RGF merged, and these 3 types of RGFs with 3D coordinates of joints are enough to get high quality neighborhood.

**Figure 21:**
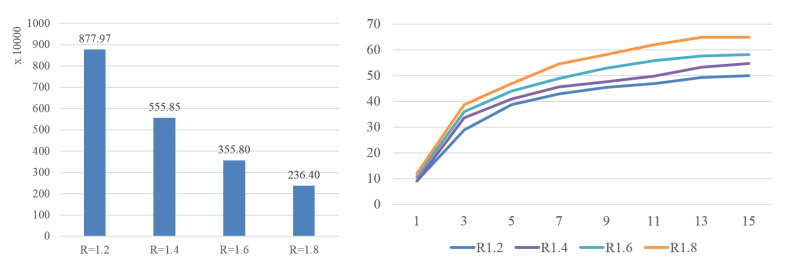
**Left**: number of quartets with different *R* on set #2. **Right**: average distance of the phylogenetic tree generated with different *R*.

As mentioned before, threshold *R* controls the number of reliable quartets. The left of Figure 21 shows the number of quartets with different *R* on set #2. When *R* is 1.2, it arrives at around $10^{6}$ quartets and it decreases when *R* increases. However, the performance decreases correspondingly, as shown in the right of Figure 21. The reason is the small number of quartets cannot provide enough information for WQMC to deduce the fine relationship of poses. However, when *R* is too small, there will be too many quartets which affects the efficiency of algorithm. In our experiments, we set *R* to 1.2 and achieved a fairly good performance. Additionally, the running time complexity of generating quartets is quadratic, while the WQMC algorithm exhibits a performance that is close to O(N∗log(N)), where *N* is the number of poses in the input set.

### 4.3. Evaluation of Pose Context Auto-Encoding

**Figure 22:**
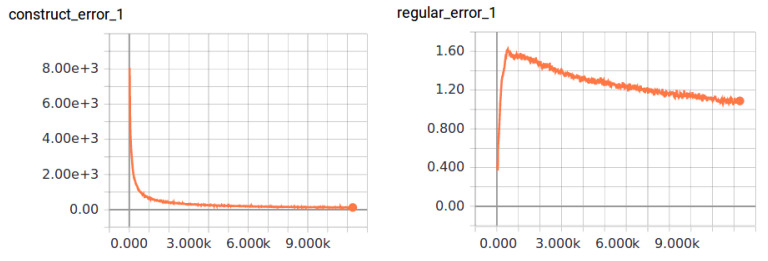
Change process through training, **left**: average motion reconstruction error, **right**: regularization term of Euclidean distance on latent space of adjacent poses.

The proposed biLSTM-based auto-encoder is implemented by TensorFlow on the platform with GPU Tesla P100. There are 2605 motion clips in CMU’s motion capture database. We sub-sampled this data to 30 frames per second and separated them into segments with 160 frames, about 5 s. For each segment, we appended 30 frames to both ends as context. This results in total 7915 segments. It needs about 10,000 epochs for training. The learning rate is set to 0.001 in the first 1000 epochs, then decreases linearly to minimum 0.0001 at epoch 8000. The change processes of average motion reconstruction error and regularization term of Euclidean distance on latent space of adjacent poses through training are shown in the left and right of Figure 22.

**Figure 23:**
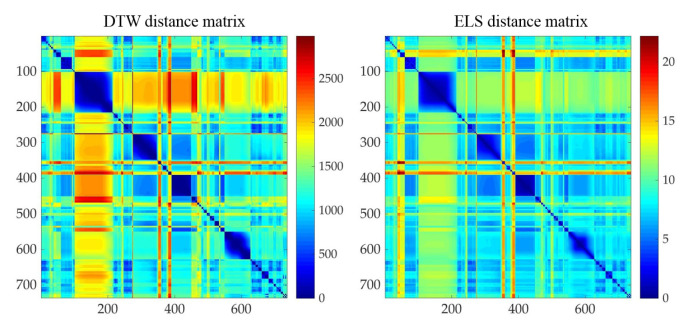
Distance matrices of motions relevant to set #1, **left**: DTW distance, **right**: ELS distance.

There are 736 motions whose poses or their cluster exemplar poses belong to set #1. To verify the validity of using Euclidean distance on latent space as motion distance measure, we calculated both dynamic time warping (DTW) on 3D coordinates of joints and Euclidean distance of latent representation *z* for these motion segments (call them DTW distance and ELS distance respectively for short in the following). Then we ran the Spearman rank correlation between two distance measures. The correlation coefficient is 0.9080, and there exists the significant correlation. The plotted distance matrices are illustrated in Figure 23.

**Figure 24:**
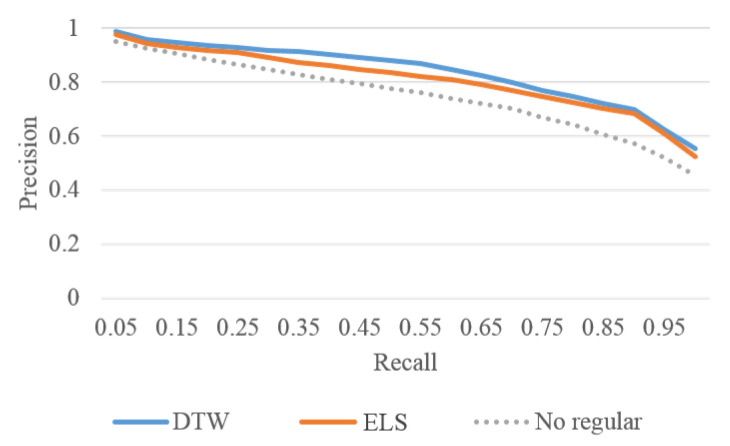
Recall-precision curves of three measures, i.e., DTW distance, ELS distance and ELS distance achieved without the regularization term.

To further evaluate the effectiveness of the proposed biLSTM-based auto-encoder, KNN-based motion ranking is used for motion exploration when user wants to find more motions similar to his or her interests. We first applied AP cluster with DTW matrix on 736 motions and got initial 72 motion categories, based on which we then manually labelled 20 categories as ground truth. The number of samples of categories varies from 18 to 52. We randomly selected 10 motions from each category as queries, and ran totally 200 query sessions on the collection of 736 motions. The average recall and precision are used as the criteria to evaluate the ranking performance. Recall is defined as the ratio of the number of relevant motions in the returned motions to the total number of relevant motions in the collection. Precision is defined as the ratio of the number of relevant motions in the returned motions to the total number of returned motions. Figure 24 shows the average recall-precision curves.

The *X* axis in Figure 24 is the recall ratio. The *Y* axis is the precision and it decreases with the increase of recall. The blue line is the performance of DTW distance and the orange line is the performance of ELS distance. The max precision difference of these two measures is 0.0467 when recall is 0.55. The precision of DTW is 0.8689, while that of the proposed method is 0.8222. From Figure 24, it can be seen that the performance of ELS distance is very close to the performance of DTW distance. In Figure 24, the dotted line is the performance of ELS distance which achieved without the regularization term. The effective of the regularization term is clear and gets 6.24% gain when recall is 0.55 ( 0.8222 vs. 0.7598).

**Figure 25:**
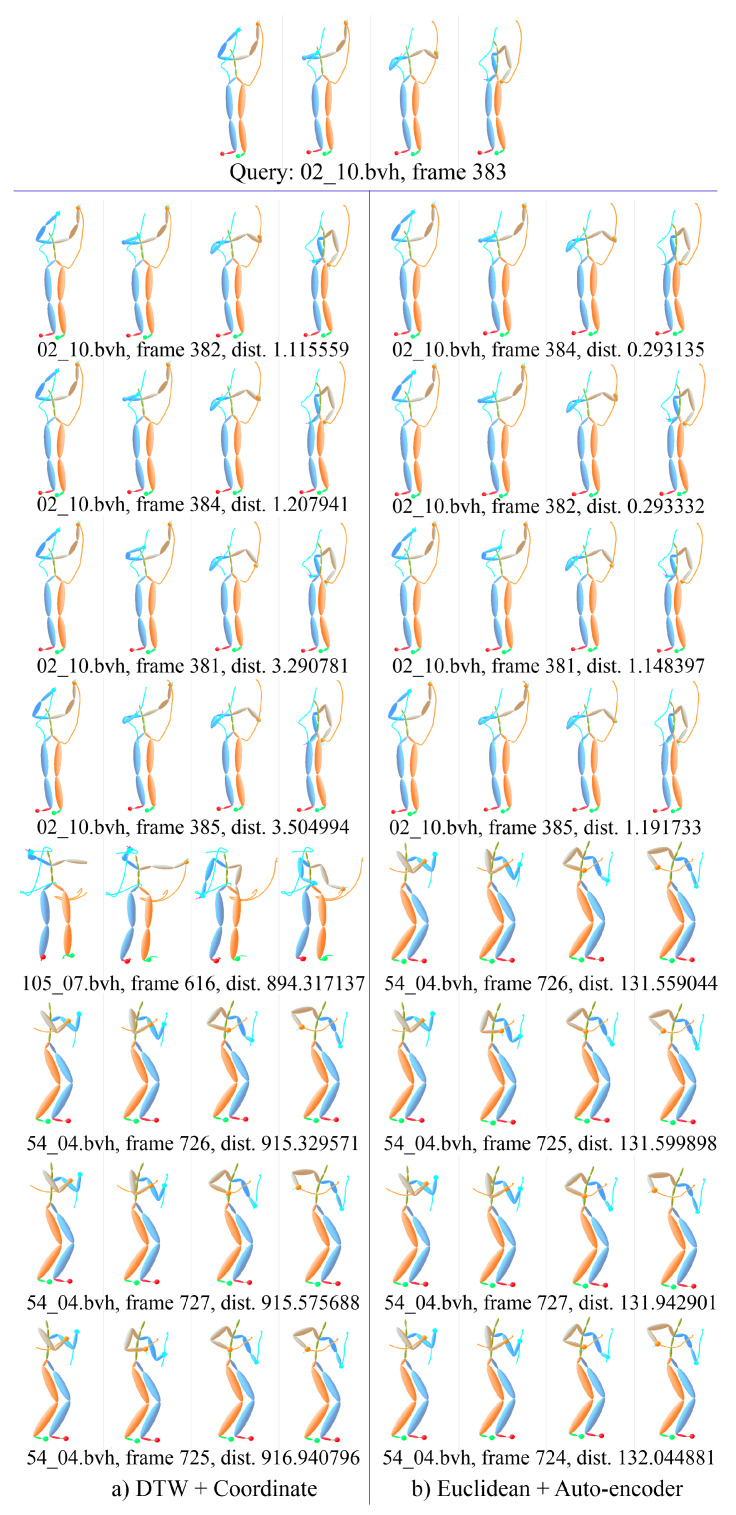
Example of KNN-based motion ranking with measure of DTW distance and ELS distance, the interesting motion is *wash self*.

**Figure 26:**
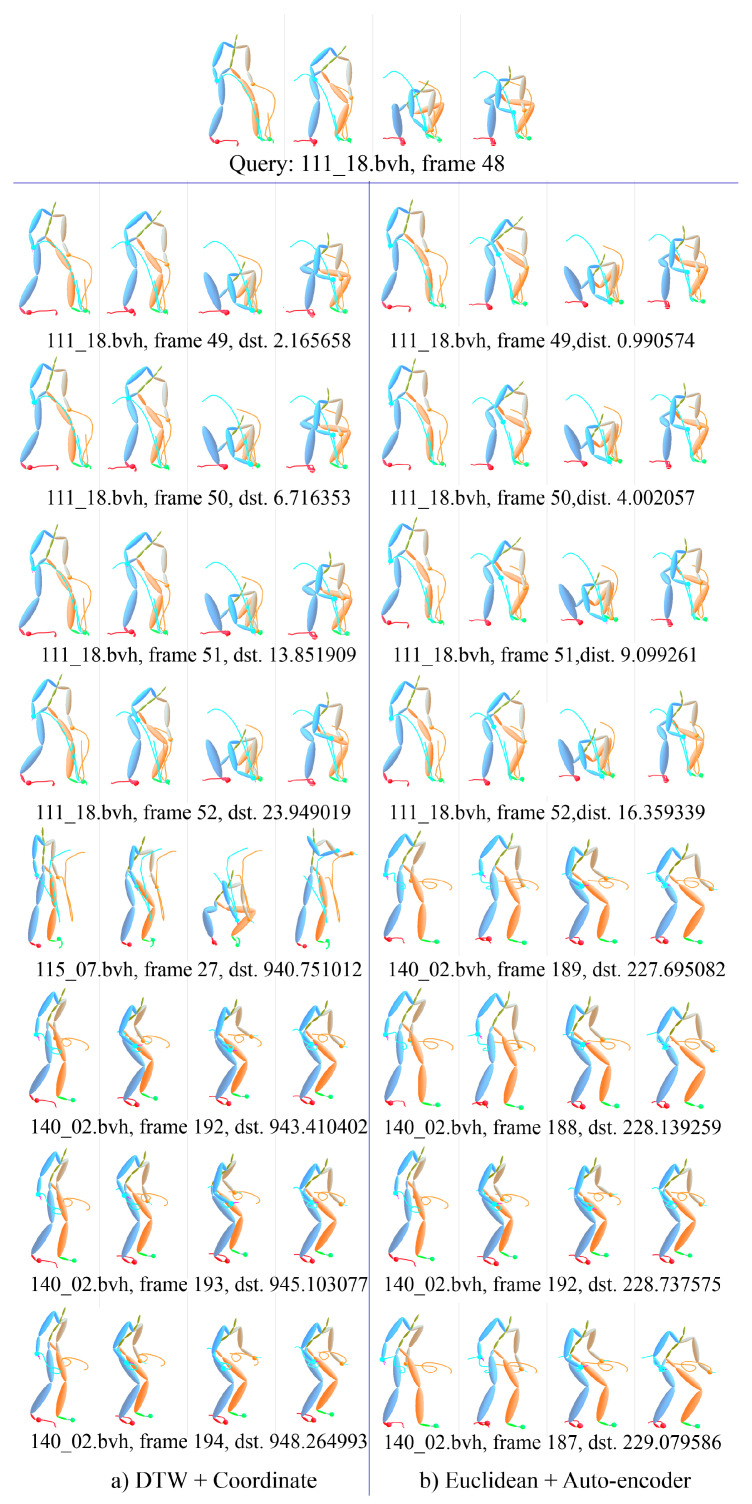
Example of KNN-based motion ranking with measure of DTW distance and ELS distance, the interesting motion is *pick up*.

Figure 25 gives a detail example of motion ranking. Each motion is presented with 4 frames, i.e., the first, the end and two middle frames. The first line is the interesting motion *wash self*, the subject of which is 02 and trial is 10 in CMU’s motion capture database. The center frame index is 383, which means the segment starts from 353 frame and ends at 413 frame, total 61 frames. The left under the interesting motion is the nearest motions by KNN with the measure of DTW distance. The right is the nearest motions by using ELS distance. It can be seen that only the motion of 105_07.bvh in the left is not in the right, and other motions just have slight difference in the position. Figure 26 gives another example of the motion ranking. The interesting motion is *pick up*, the subject of which is 111 and trial is 18. Similar to the previous instance, only the motion of 115_07.bvh in the left is not in the right.

**Figure 27:**
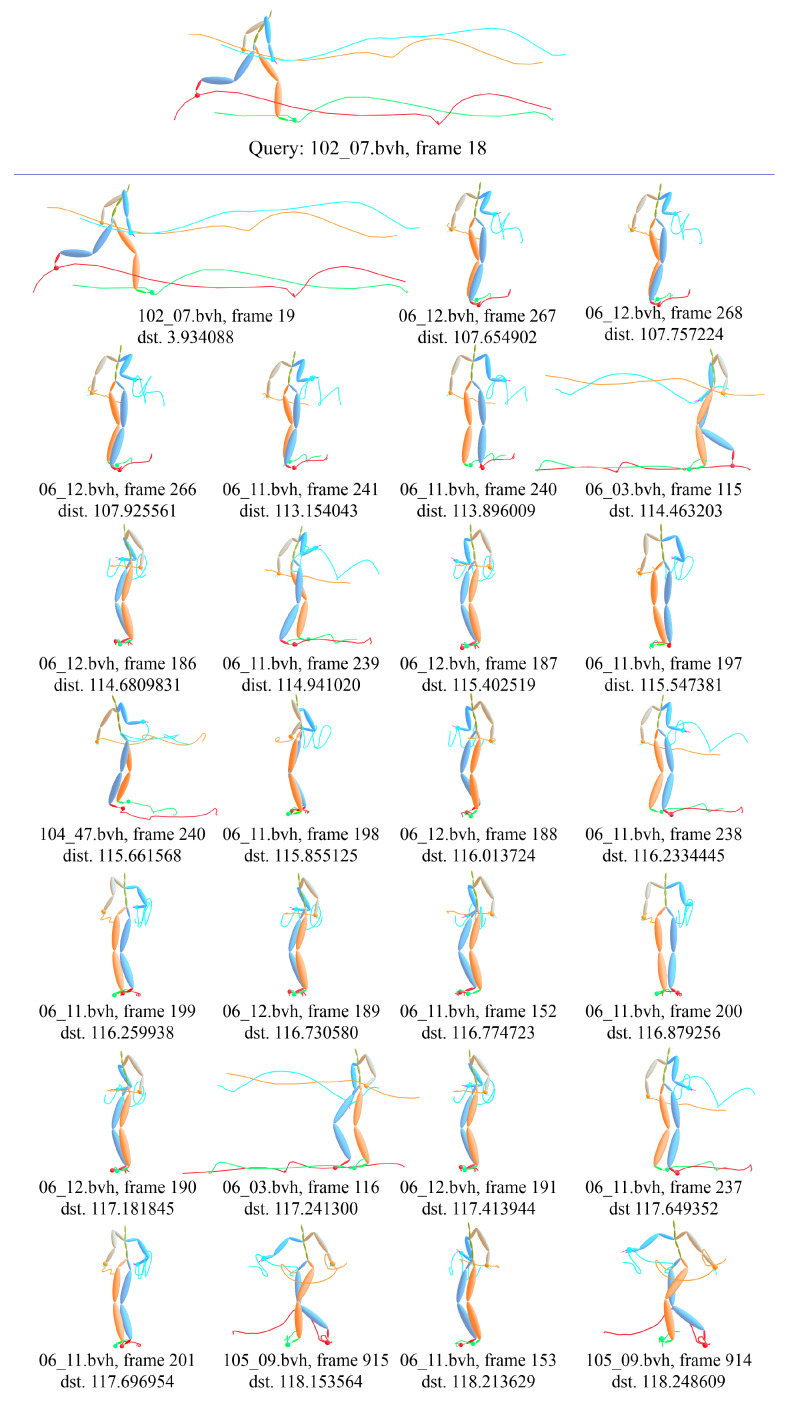
Example of KNN-based motion ranking with ELS distance, the interesting motion is *run wide left of basketball movement*.

The above two examples give 8 motions nearest to the interesting one. Figure 27 shows a detail example with 27 nearest motions based on KNN with the proposed ELS distance. The first line is the interesting motion *run wide left of basketball movement*, the subject of which is 102 and trial is 07, and the center frame index is 18. The 27 nearest motions scatter over *run wide left of basketball movement* (102_07.bvh), *forward and crossover dribble of basketball movement* (06_03.bvh, 06_11.bvh, 06_12.bvh), *drunk walk* (105_09.bvh).

The first two examples indicate that the proposed ELS distance can match DTW to measure very similar motions, while the last example shows the proposed measure also can catch similar motions with style variation. The above experiments verify the effectiveness of the proposed biLSTM-based auto-encoder. Notice the time complexity of DTW is O(m∗n∗p), while the time complexity of our method is only $O(p^{\prime })$. For instance, on Intel Core^*TM*^i5 platform with the fastDTW package of Python, calculating the distance from one action to 736 actions takes an average of 14.7 seconds, while our method has a response time of less than 0.1 seconds, which makes it possible to support the user to perform online motion exploration such as motion ranking and clustering for fast motion search. A concrete example will be presented in the next section.

## 5. Prototype of Our System

**Figure 28:**
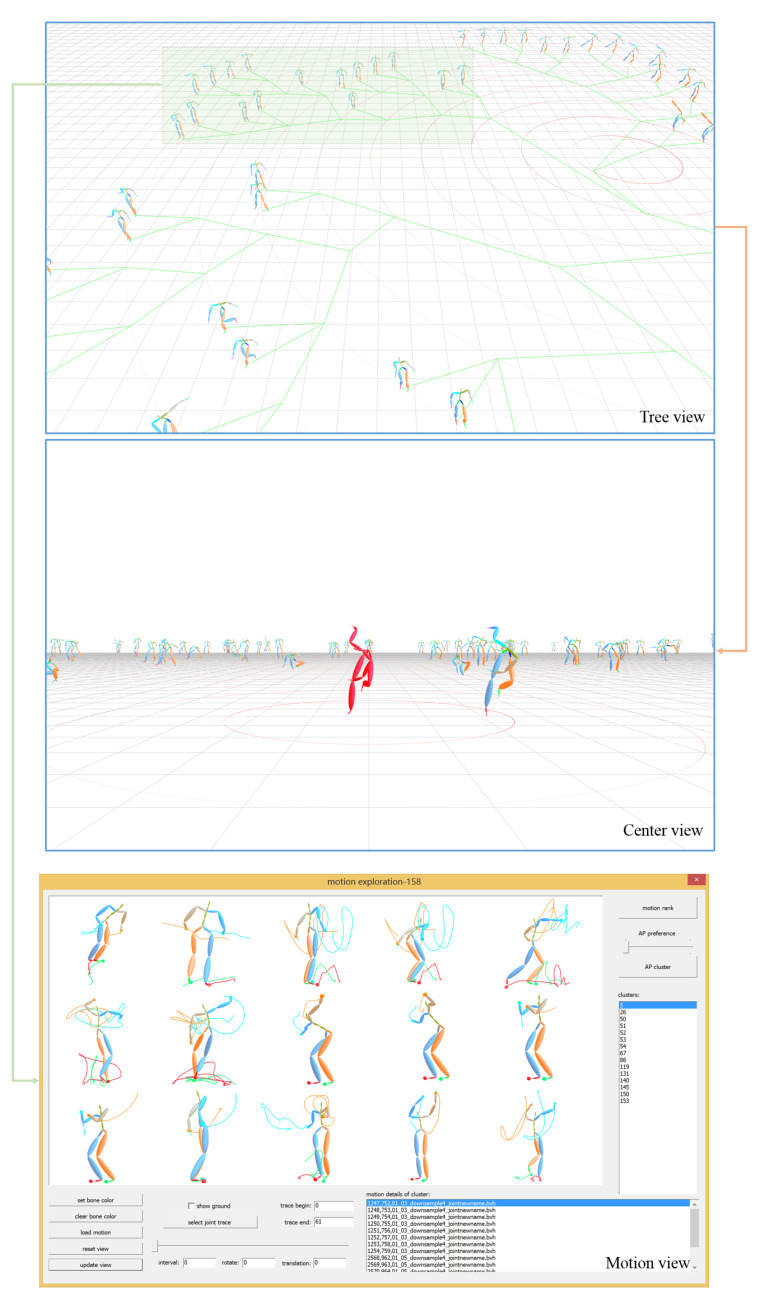
Prototype system for pose browse and motion exploration, which has three main 3D views, i.e., tree view, center view and motion view.

We have realized a prototype system on CMU’s motion capture data. The prototype system has three main 3D views as shown in Figure 28. The top is the tree view showing the neighborhood of all representative poses of clusters, which allows user to perform fast browse to understand the overview of the data collection and locate interesting poses quickly. The middle is the center view, in which the user selected poses are surrounded by other poses whose locations are determined by the edge counts to the selected one. It provides another means for user to implement quick comparison. The bottom is the motion view, which enables the user to perform fast online motion exploration with analysis tools for ultimate motion search.

The user can quickly switch among these three views. The top of Figure 28 shows set #1 containing 100 representative poses in the 3D space (as shown in Figure 15 in 2D space). After selecting an interesting pose in the tree view, the user can switch to the center view and the rest of the poses are automatically repositioned to form a circle chart around the selected one, as shown in the middle of Figure 28. When locates the interesting branch in the tree view, as shown in the top of Figure 28 which contains 16 representative poses, the user can switch to motion view to further investigate the relevant 158 motions whose poses or their cluster exemplar poses belong to the enclosed region. KNN-based motion ranking and AP-based motion clustering are two convenient analysis tools for motion exploration. In the bottom of Figure 28, 158 relevant motions are grouped into 15 clusters with AP. The first motion of each cluster is shown in the view. The user also can adjust the preference of AP to refine the cluster results. According to the response from the investigated user, our prototype system provides a convenient interactive environment for the user to perform pose browse and motion exploration.

## 6. Conclusions and Future Work

In this paper, we present a novel approach to organize the collection of motion capture date for pose browse and motion exploration. The keys of our method include data abstraction with AP to obtain representative pose of clusters, neighborhood construction with phylogenetic tree to organize the similar isolated representative poses to be adjacent with each other, and biLSTM auto-encoder to encode the pose context into the compact latent space to support online motion exploration. However, there are still several limitations which may spark future research:

(1) Our approach uses several common distance measures of pose, but fuses them in an effective manner. However, the final phylogenetic tree relies ultimately on the effectiveness of the measure of pose, and more measures which consist with the similarity of human conception are needed to enhance the performance for neighborhood construction.
(2) The traditional hierarchical clustering algorithms used for motion capture data [10] are greedy while our method performs global analysis. However, the generation of weight quartets is independent of the construction of phylogenetic tree, which leads to large number of quartets are generated but many of them may not be necessary. Move effective method is needed to fuse these two processes to enhance the efficiency.
(3) The confliction of quartets generated by different features is solved by WQMC and can be regarded as a form of voting. In fact, this confliction also can be settled by active learning. We can first find violated quartets and rank the triples contained in the violated quartets according to the inconsistence degree. Then, the user labels the recommended triples and the system generates a new phylogenetic tree with the refined quartets. The above gives an outline of one possible solution, but further in-depth study need to be conducted.
(4) We provide KNN-based motion ranking and AP-based motion clustering with the proposed Euclidean distance on latent space for motion exploration. These two analysis tools are necessary for fast motion location on the coarse level. More methods with their visualization techniques are needed for quantitative detail motion analysis.
